# Signatures of medical student applicants and academic success

**DOI:** 10.1371/journal.pone.0227108

**Published:** 2020-01-15

**Authors:** Tal Baron, Robert I. Grossman, Steven B. Abramson, Martin V. Pusic, Rafael Rivera, Marc M. Triola, Itai Yanai

**Affiliations:** 1 Institute for Computational Medicine, New York University Grossman School of Medicine, New York, New York, United States of America; 2 Institute for Innovations in Medical Education, New York University Grossman School of Medicine, New York, New York, United States of America; 3 Department of Radiology, New York University Grossman School of Medicine, New York, New York, United States of America; 4 Department of Medicine, New York University Grossman School of Medicine, New York, New York, United States of America; Florida Agricultural and Mechanical University, UNITED STATES

## Abstract

The acceptance of students to a medical school places a considerable emphasis on performance in standardized tests and undergraduate grade point average (uGPA). Traditionally, applicants may be judged as a homogeneous population according to simple quantitative thresholds that implicitly assume a linear relationship between scores and academic success. This ‘one-size-fits-all’ approach ignores the notion that individuals may show distinct patterns of achievement and follow diverse paths to success. In this study, we examined a dataset composed of 53 variables extracted from the admissions application records of 1,088 students matriculating to NYU School of Medicine between the years 2006–2014. We defined training and test groups and applied K-means clustering to search for distinct groups of applicants. Building an optimized logistic regression model, we then tested the predictive value of this clustering for estimating the success of applicants in medical school, aggregating eight performance measures during the subsequent medical school training as a success factor. We found evidence for four distinct clusters of students—we termed ‘signatures’—which differ most substantially according to the absolute level of the applicant’s uGPA and its trajectory over the course of undergraduate education. The ‘risers’ signature showed a relatively higher uGPA and also steeper trajectory; the other signatures showed each remaining combination of these two main factors: ‘improvers’ relatively lower uGPA, steeper trajectory; ‘solids’ higher uGPA, flatter trajectory; ‘statics’ both lower uGPA and flatter trajectory. Examining the success index across signatures, we found that the risers and the statics have significantly higher and lower likelihood of quantifiable success in medical school, respectively. We also found that each signature has a unique set of features that correlate with its success in medical school. The big data approach presented here can more sensitively uncover success potential since it takes into account the inherent heterogeneity within the student population.

## Introduction

The medical school admissions process is a resource-intensive challenge for all concerned, with many more applicants than available positions. According to the Association of American Medical Colleges (AAMC), 53,042 individuals applied to US medical schools in the 2016–2017 application cycle, and of those, 21,030 ultimately matriculated. At our NYU Medical School, 7,679 applicants sought a place in a class of 120 matriculating students during that same admissions cycle. Given this highly competitive process, it can be challenging for medical school admissions committees to identify and select those applicants that are most promising and that best align with the stated missions of the individual school [1]. During some stages of this process, candidates may be considered collectively as a homogeneous population for whom generalized cutoffs for performance in standardized tests and undergraduate grade point average (uGPA) are used [2–13].

This ‘one-size-fits-all’ approach, however, neglects the common sense observation that individuals show distinct patterns of achievement and follow diverse paths to success [14]. An alternative approach to understanding the relationship between undergraduate performance of students and their success as medical students would thus account for the known heterogeneity of this population. In other words, distinct clusters of students may be delineated in terms of their pattern of achievement [15]. With the rise of data-driven approaches [16–20], it may now be possible to more efficiently detect such clusters. We therefore reasoned that instead of studying students as one group, the machine learning derivation of distinct clusters might allow different factors to emerge as being predictive of success.

To test this idea, we used intensive computational approaches to investigate a large dataset of student records including admissions data to medical school and success in their subsequent education. As a proxy for success in medical school we analyzed an extensive set of measures including exam scores and admission to an academic honor society. While this measure of academic success does not necessarily correlate with professional success and patient satisfaction, we sought to elucidate patterns that could be helpful in adjusting the support students receive on their journey through medical education.

## Materials and methods

### Ethics statement

Ethical approval has been granted by the New York University Grossman School of Medicine Institutional Review Board (i17-00533), approved 5/2/2017. The data is fully de-identified and the requirement for informed consent was waived.

### Demographic and overview

The overall dataset was made up of the medical application records of 1,088 students dating between 2006 and 2014. For most of these students we also had data on their subsequent academic success in medical school. One student was inexplicably missing MCAT data, while 141 students were still active students and therefore lack some of the outcome data, resulting in a subset of 946 student records with complete dataset dating between 2006 and 2013 that were used for analysis (S1 Table, see S1 Supplementary Note). These comprise 53 features and 8 quantitative academic success outcomes for each student. The application features and academic outcomes are described in S1 and S2 Tables, respectively, including the effect size differences (Cohen’s d) between the training and test groups (For further explanation on features, see S1 Data).

### Machine learning approach to data analysis

**Fig 1:**
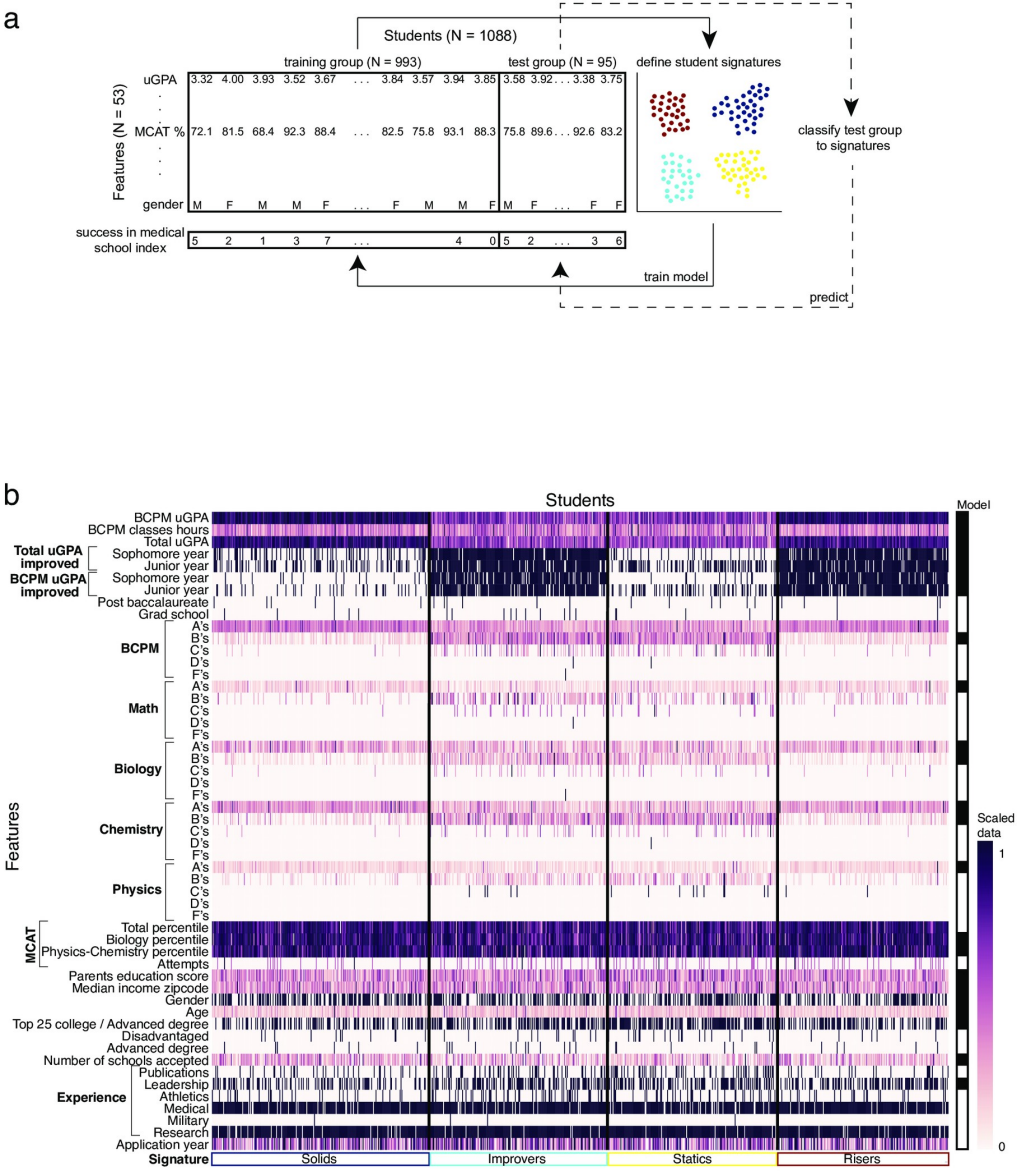
Medical school students signature approach and admissions data. (a) Schematic of the signature classification approach. Our dataset comprises 1,088 students from NYU School of Medicine accepted from 2006 to 2014. For each student 53 features are known, as well as 8 ‘outcome’ features documenting success during medical school for 946 of those students. In the present work, we define signatures using the training group and use them to train a model including the signature to better predict success in medical school. Finally, we deploy the model on the test group to predict their success in medical school. (b) Student records from NYU medical school for 2006–2013. The heat-map indicates the scaled values for each of the 53 features (rows) and 946 students (thin columns). See S1 Data for the full dataset. The right-most column indicates the 23 features that were selected for the clustering model (in black).

The general machine learning approach includes delineation of the specific dataset, feature engineering and delineation of training and test sets [21]. Our initial dataset was in the form of a table where rows correspond to applicants and columns correspond to features; *i*.*e*., the variables known about the applicants, such as GPA score, age and gender (Fig 1a). Feature engineering includes the construction of additional features based upon the initial raw variables. For example, in our dataset we defined an improvement feature—or *trajectory*—between consecutive years based upon the uGPA data of the applicants (1/0; applicant improved or not, respectively, see S1 Data). Feature engineering also includes the integration of different data types for increased algorithmic performance by dichotomizing continuous features. Finally, the dataset was randomly split into training and test groups accounting for 90% and 10% of students, respectively (see S1 Supplementary Note).

### Dataset preparation

We collected the application records of 1,088 students matriculating to NYU Grossman School of Medicine between the years 2006–2014 (from the American Medical College Application Service and the specific NYU School of Medicine forms). These de-identified data records included 53 features for each student including the undergraduate GPA (uGPA), MCAT scores, gender, as well as socio-economic features (see Fig 1b, S1 Table and S1 Data). Some of the features were discrete, such as gender and possession of an advanced degree(s), while others were continuous, such as uGPA, age, and number of MCAT exam attempts [2,5]. We integrated both types of features by dichotomizing the values of each continuous feature across the students according to the feature-specific median: values lower or equal to the median were set to zero, while the remaining were set to one [22]. This approach was deployed separately for the students of each application year, to account for possible changes in admissions criteria across years thus controlling for cohort effects. Thirteen features had no variance in at least one of the years under study, resulting in their exclusion from further analysis, since dichotomization could not be computed. Prior to analysis, we set aside a randomly selected ‘test group’ consisting of 10% of the students for later testing, and we referred to the remaining 90% as the ‘training group’. The large fraction of students included in the training group was due to the relatively small number of students with available data.

### K-means clustering of applicants

To search for clusters of students based upon their application data, we used a feature selection approach to identify the set of features that yield the most coherent clusters. This process does not include any of the components of the success index, which is used later to determine to what degree clusters based on data available at admissions have implications for later medical school success. We defined an index score to summarize the tightness—as measured by the squared distances from the cluster centroids (S1 Fig)—and evenness of the clusters, and the mean variance and redundancy of a given set of features (see S1 Supplementary Note). Using a custom-made “greedy” algorithm [23], we converged upon a set of 23 features that are most informative for robust student clusters (Fig 1b, right bar). We then defined four groups of students in the data space of the 23 informative features by K-means clustering [24]. By plotting the sum of squared distances from cluster centroids as a function of number of clusters, we limited the number to four, as the second differential is greatest at this point (S1 Fig). To visualize these clusters we used dimension embedding on the table of students and the 23 informative features using t-SNE [25].

### The academic success index

For 946 of the 1,088 students we had data on the academic success of the students during their medical school training. The total outcomes that we had access to are clerkship honors (the overall number, the number of clerkship fails, and the number of low scores), entry into the Alpha Omega Alpha Honor Medical Society (AOA), US Medical Licensing Examination (USMLE) Step 1 and 2 scores, and shelf exam scores (the number above 90 and the number below 65). We sought to create an aggregate success index based upon these available seven ‘outcomes’. Since outcomes were both binary and continuous we dichotomized each using the same approach as for the application features described above. The aggregate success index was then calculated as the sum of all seven available binary outcomes, resulting in a score ranging from 0 (worst) to 7 (best).

### Logistic regression to predict academic success from applicant data

Finally, we asked if we could demonstrate that the signature is an important feature by determining if it would improve the prediction of academic success. Specifically, to assess whether the four clusters explained variability in the success index beyond that achieved with the raw data, we determined if predicting the success index from the base application features is differentially more accurate when the signature of each student is added to the model. For this we used the following two approaches for predicting the success index: 1. using the application features alone, and 2. including also the signature assignments. To produce a valid and robust predictive model we further compressed the success index into a three-level success score. This was done by scoring low performing students (original success index scores of 0, 1 and 2) with 0, medium performing students (original success index scores of 3 and 4) with 1, and high performing students (original success index scores of 5, 6 and 7) with 2. Compression is helpful in that every level of the success score relates to a higher number of samples, which leads to decreased error rate of the classifier [26]. We used a 3-fold cross validation procedure to find an optimized logistic regression model, and fitted it on the features of the training group, using the compressed success score as the target variable. We deployed this model on the test group to predict the compressed success score of these students. In parallel, we fitted a second optimized logistic regression model as above but also include the signature assignment as an additional feature. To infer the cluster of the students in the test group, we matched the nearest K-means cluster delineated by the training group. The Likelihood Ratio (LR) test was performed on the two fitted models using the training data to compare the goodness of fit of the two models.

## Results

### Signatures of medical school students

**Fig 2:**
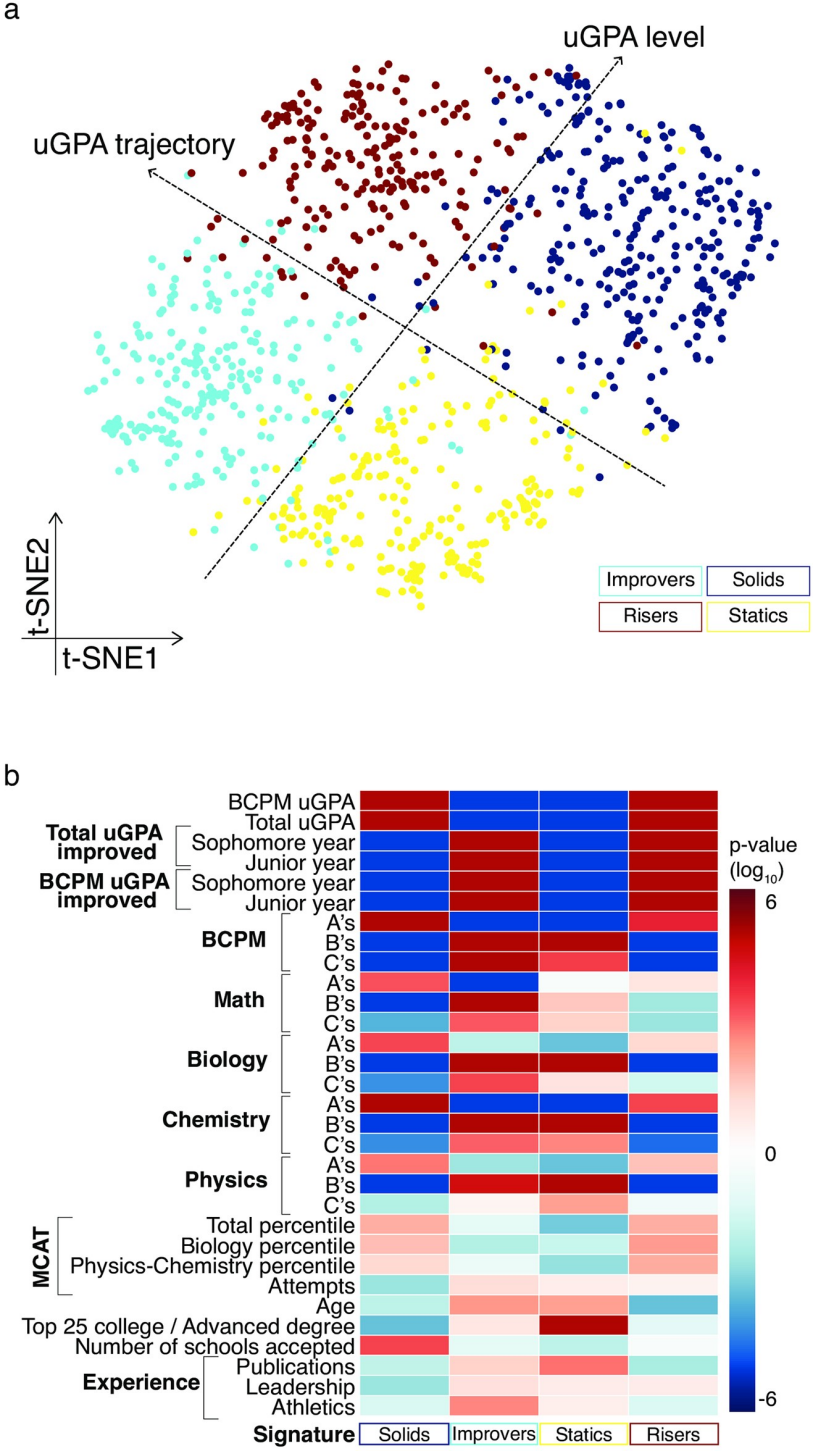
Medical school students can be classified into four signatures based on admissions data. (a) Embedding the 23-feature-space student data into a two-dimensional space, using t-SNE analysis [25], allows us to visualize similarities among students, and reveals four clusters. Each dot indicates a student and is colored by the K-means clustering (see Materials and methods). The different colors correspond to the four different signatures. (b) Characteristics of the signature features. For each signature, the heat-map indicates the enrichment (-log_10_ *P*-value) for each of the features shown (see Materials and methods). Only those 31 out of 53 features with a significant value (*P* < 0.01) in at least one signature are shown.

Feature selection and clustering on our database of applicant data yielded four signatures with two main distinguishing attributes: the absolute level of the uGPA and its trajectory throughout the undergraduate studies of the student (Fig 2a). For each of the four signatures, we could systematically examine the distinguishing features (Fig 2b), according to which, we named the signatures as the ‘statics’, ‘solids’, ‘risers’, and ‘improvers’. The ‘statics’ are statistically over-represented for students from undergraduate schools that rank in the top 25 on the U.S. News & World Report rankings of undergraduate universities. In other words, the group of students defined as statics (Fig 2a, yellow circles), has significantly more students from top undergraduate schools than expected relative to a randomly selected group of the same size (67.2% vs 52.4%; 95% CI difference [7.7%, 22.1%]). This group has relatively lower uGPA and tends not to improve during their undergraduate years. The ‘solids’ and the ‘risers’, in contrast, have the best uGPAs. They are distinguished from each other in that the ‘risers’ showed a trajectory of improvement in uGPA throughout their undergraduate studies. On average, 89.7% of risers improved their uGPA from freshman to sophomore or sophomore to junior years of college, while only 31.5% of solids on average showed a trajectory of improvement during the same period. Finally, the ‘improvers’ also showed an upward trajectory, though their uGPAs were relatively lower than those of their medical school peers (Fig 2b). Other interesting aspects of the signatures are that: 1. the solids were accepted into more medical schools (solids 3.7 vs all others 3.3; 95% CI difference [0.171, 0.625]), 2. the improvers are statistically over-represented for athletes (23.8% vs 16.1%; 95% CI difference [2.4%, 12.6%]), 3. the statics have the highest proportion of students with academic publishing experience at the time of application (27.7% vs 18.7%; 95% CI difference [3.6%, 14.4%]), and 4. the risers are the youngest (21.296 vs 21.622; 95% CI difference [-0.543, -0.126]) (*P* < 0.01, for all; see S1 Data for signature means and standard deviations). From this analysis, we concluded that distinct medical student signatures may be delineated from the application data, each with distinguishing properties.

### Success across the signatures in medical school

**Fig 3:**
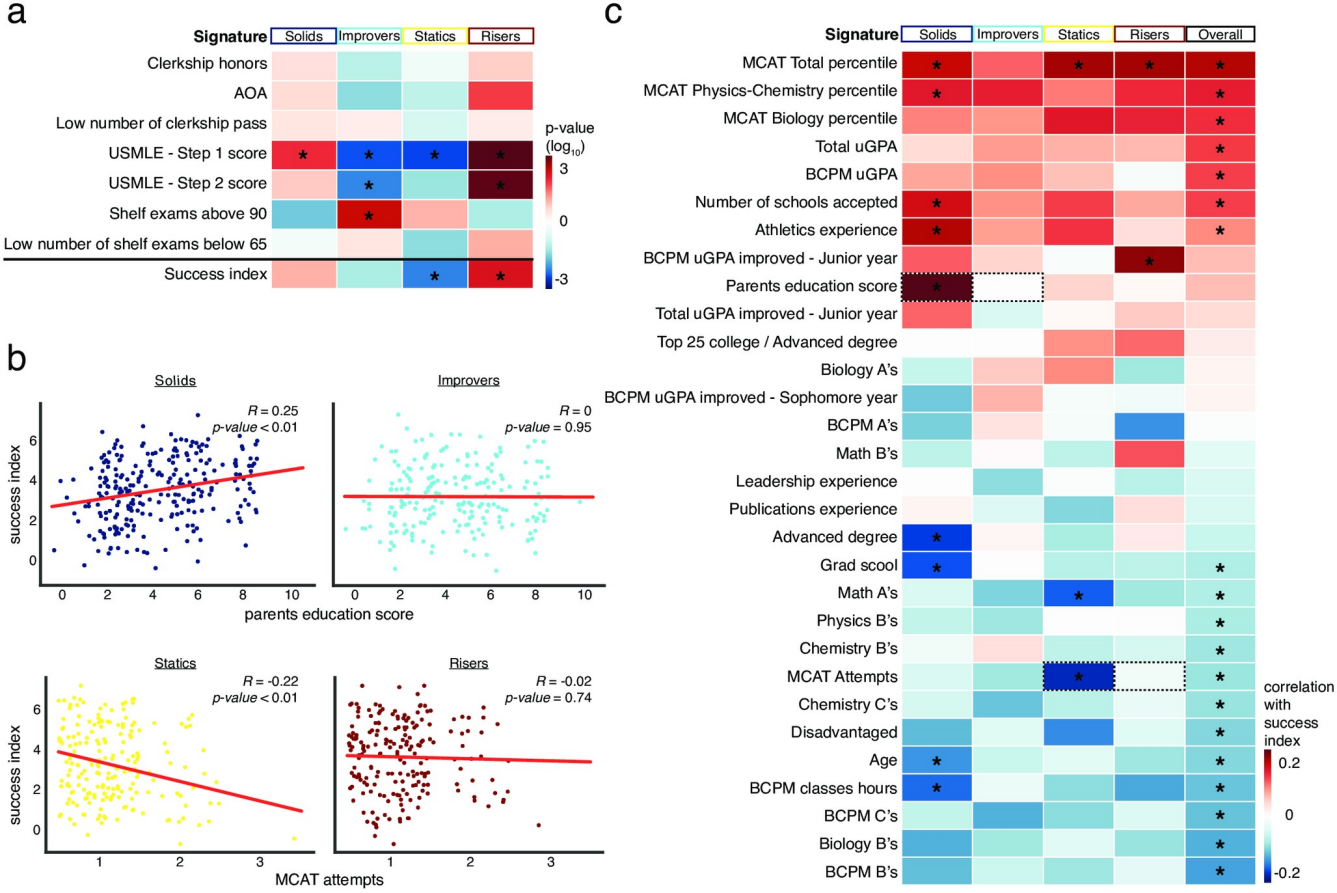
The relationship between student signatures and their success in medical school. (a) Signatures show different levels of success. For each signature, the heat-map indicates the significance of the average level of a success measure relative to the average level in a randomly selected group of the same size. The ‘success index’ shown in the last row is a combined score of the above outcomes (see Materials and methods). Stars indicate *P*-value less than 0.01. (b) Parent education score and the number of MCAT attempts predict success differently across signatures. The two upper plots show the correlation between parent education score and the success index for solids (left) and improvers (right). The two bottom plots show the correlation between the number of MCAT attempts and the success index for statics (left) and risers (right). The points were jittered to better show the points. (c) Different features differentially predict success across signatures. The heat-map summarizes the correlations between the features and signatures. Only features with a minimal absolute Pearson correlation coefficient of *R* > 0.1 in one of the signatures or overall are shown. Stars indicate *P*-value less than 0.01.

We next asked if the students of the four signatures performed differently during their medical school training, according to the success index (see Materials and methods). This analysis is possible since the signatures were derived without association to success factors. For each student, our dataset includes information on clerkship scores, entry into the Alpha Omega Alpha Honor Medical Society (AOA), US Medical Licensing Examination (USMLE) scores, and shelf exam scores (S2 Table). We found that the signatures showed significant differences across several measures of success (Fig 3a and S1 Data). For the USMLE Step 1 scores, the improvers and the statics significantly underperformed (236.2 and 236.0, respectively vs 239.4; 95% CI difference [-5.47, -0.95] and [-5.74, -1.1], respectively; *P* < 0.01, for both), while the solids and risers performed significantly better (241.8 and 243.0, respectively vs 239.4; 95% CI difference [0.39, 4.46] and [1.36, 6], respectively; *P* < 0.01, for both). To study medical school performance, we created a ‘success index’ that summarizes the seven outcome measures. Examining the success index across signatures, we found that the risers and the statics have significantly higher and lower success indices, respectively (Fig 3a and S1 Data), while the improvers and solids did not differ from the overall average.

Admission procedures seek to predict success in medical school based upon the features used in our model. Since our results indicate the existence of signatures, we thus asked if different signatures show distinct features correlating with success (since these were not used to define the signatures). Fig 3b shows an example of this analysis for the ‘parents education score’ feature: solids signature show a correlation between this feature and success in medical school. However, for the improvers there is no such correlation. Thus, by focusing on a particular subset of the student population (the solids) we are able to identify a feature that, among the solids alone, correlates with the success of these students. As a second example, the ‘MCAT attempts’ feature is negatively correlated and not correlated with success in the statics and the risers, respectively (Fig 3b). More generally, Fig 3c indicates those features with an absolute correlation coefficient of at least 0.1 with the success index in one or more signature, or the overall set of students. Some features positively or negatively correlate with success in a similar way across all signatures, while other features show signature-specific correlations. For example, for the risers, the uGPA in biology / chemistry / physics / and math (‘BCPM uGPA’) is not predictive of success, however for the other three signatures it is. Conversely, the improvement of the risers in these courses (‘BCPM uGPA improved—Junior year’) is correlated with their success, though it is not correlated with the success of the statics (Fig 3c). Thus, each of the four signatures has a unique set of features correlating with success in medical school, demonstrating the avenues to success available for the students across signatures.

### Do signatures improve predictions of differential success?

**Fig 4:**
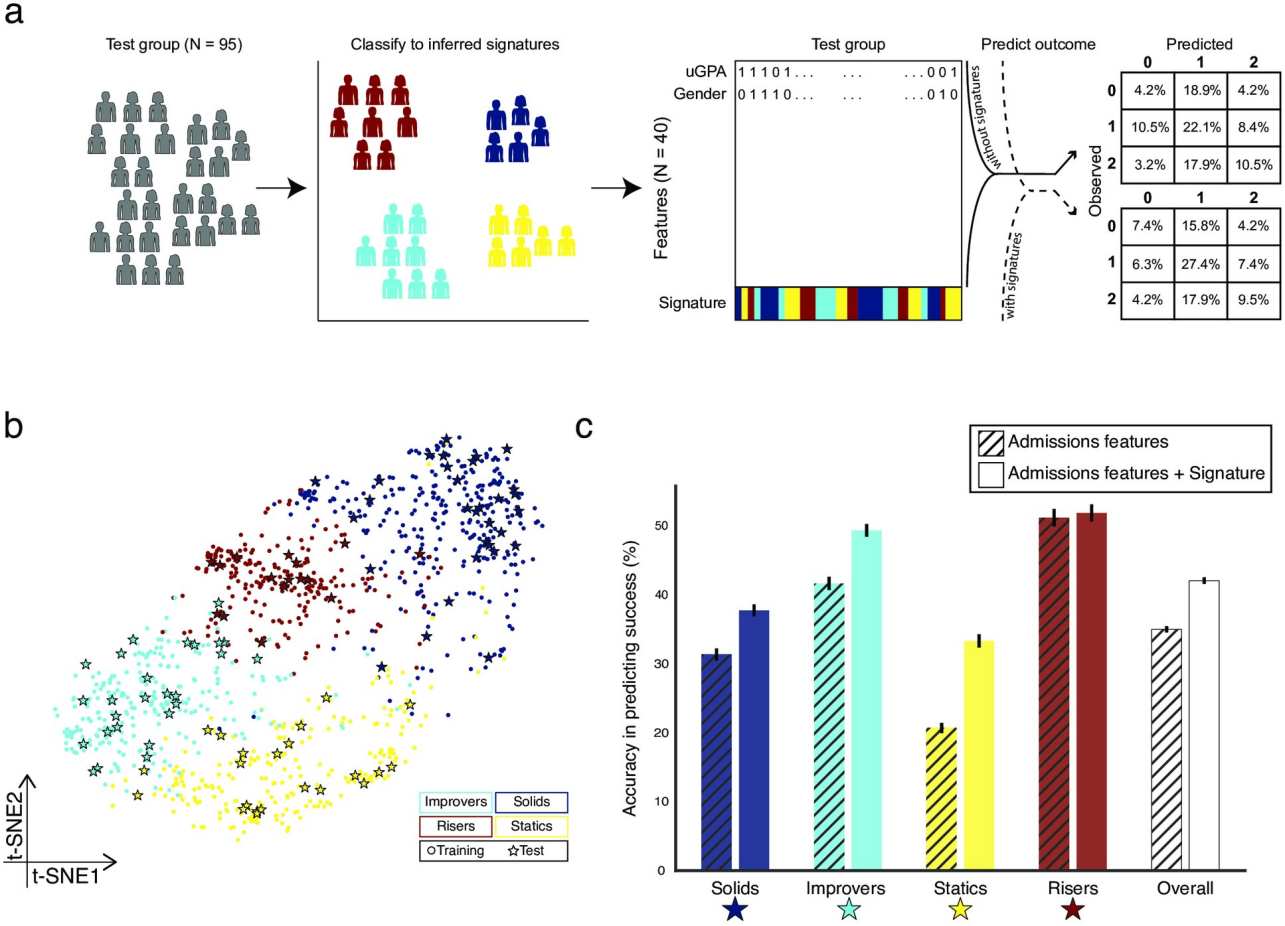
Prediction of success increases with inclusion of signature inference. (a) Schematic of the approach for prediction. Students from the test group (Fig 1a) were classified to signatures. A logistic regression model was then deployed to predict success using two models differing only in whether they also include the signature classification as a feature. The model including the signature classification as a feature showed increased predictive power. The two confusion matrices present the actual logistic regression results. (b) Dimension embedding on all students—clustering training and test groups—using t-SNE. Note that the students in the test group (stars) are well classified into the originally defined signatures (shown in Fig 2a). (c) Predicting the outcome of students in each signature is generally improved when including the signature classification as a feature. For each signature, the bar plot shows the accuracy of predicting success for those students classified to that signature in the test group using two models (with and without the signature classification, see Fig 4a and Materials and methods). Error bars indicate standard error according to bootstrap analysis.

If our delineated signatures differentially correlate with performance in a robust manner, we would expect that the classification of a student to one of the four signatures leads to better prediction of success in medical school. To test this, we turned to our ‘test’ group that was not used to define the signatures (Fig 1a). We classified each of the 95 students in the test group to one of the defined four signatures (see Materials and methods and S1 Supplementary Note). Performing a dimensional embedding analysis [25] on all students—training and test groups—showed the same pattern of signatures (Fig 4b), as previously observed (Fig 2a), providing support for the robustness of the identified signatures. To predict success, we fitted a logistic regression model using 40 binary features of the training group with an adjusted three-level success score (0, 1, and 2; Fig 4a). In a second model, we added to the base model the signature classification as one additional feature. We compared the goodness of fit of these two models using a likelihood-ratio (LR) test, and found that the model that included the signature as an additional feature, fitted the data significantly better than the model that did not include the signature as a feature (*P* < 0.001). We then tested the accuracy of the fitted models on the test group and found that the model that included the signature as a feature showed improved accuracy in predicting the three-level success score from 36.8% to 44.2% (Fig 4a). Thus, by simply including one composition (or composite) feature—the signature—we found an increase in success prediction. Interestingly, the model showed different accuracies across signatures (Fig 4c). In three of the four signatures, the accuracy significantly improved when considering the signature classification. Only for the risers signature there was no difference in the accuracy of success prediction with and without using the signature as a feature (Fig 4c). Thus, while the predictive power of the model is not high, its increase with the addition of the simple signature factor—which is defined only from the data—supports its importance as a measure.

## Discussion

Analyzing our extensive dataset, we detected evidence for four distinct ‘signatures’ of students, which we named the ‘statics’, ‘solids’, ‘risers’, and ‘improvers’. Each of these signatures was found to have different properties, for example in terms of their involvement in athletics, the number of medical schools they were accepted to, and their number of publications. Surprisingly, students across these signatures differ most substantially according to the grade trajectory of their undergraduate studies. We report evidence for our signatures in terms of their subsequent success in medical school. While each signature has a different overall level of success, within each signature we also find a different set of predictors of success. Thus, each signature has a unique set of features that correlate with success in medical school. Finally, we demonstrate that when taking signatures into account, prediction of success is improved.

What value does our data-driven approach to studying the relationship between student performance and success have over traditional admission methods? By analyzing many different variables on hundreds of students using ‘big data’ techniques, we were able to detect distinct subpopulations with different predictors of success that are present within the overall student population. This work thus constitutes an example of how learning analytics may allow for the personalized education of health professions based on signals that could not be detected before the organization of data into databases accessible to advanced algorithms [27–30].

A main distinguishing factor among the subpopulations—the trajectory of undergraduate grades—was found to be an important factor for success. As an example, consider how the ‘signature’ approach differs from the traditional ‘one-size-fits-all’ approach when deciding between two student applicants where applicant A has a slightly higher GPA than applicant B. While the traditional ‘one-size-fits-all’ approach clearly prefers applicant A, applicant B might be favored by our algorithm if applicant B’s trajectory of improvement outweighs the GPA difference, taking into account the other features, such as applicant A’s higher uGPA trajectory. Thus, our big data approach can more sensitively uncover success potential by taking into account the inherent heterogeneity among the student population.

Our approach for delineating signatures within the student population has important limitations. While we studied close to a thousand students, more signatures might have been resolved with data for additional students, including the entire set of applicants, creating a richer, more diverse pool of signatures than the four we describe. The data that we present and study here are from a single highly-selective institution and thus the results require replication at others. Once the hurdles involved in gathering data from across a range of institutions is overcome, we may be able to derive a general model for predicting academic success at the level of the individual, regardless of the host institution. In particular, our dataset did not include the full population of applicants to our school, but rather those that were also accepted and matriculated. These two selection mechanisms could have biased our detected associations, and ideally future studies would study data on the full population of applicants.

While we have restricted our analysis to the quantitative aspects of the application, the non-quantitative information could be used to more accurately identify student signatures. For example, interviews [31] and other non-quantitative aspects of application, such as letters of recommendations, personal statements, and other application essays may be coded and included as features for increased signature detection. Most importantly, while our study used undergraduate academic success on quantifiable measures as our proxy for success, for medical doctors the more relevant success is ability as a resident, and ultimately a physician. With further data curation, it should be possible to achieve this long-term success parameter and consequently derive a more refined set of signatures. We note that these issues of accessible, digitized data being prioritized over that which is more difficult to encode is a more general issue in a time of rapidly evolving information systems [28]. Projects like this one raise these important issues for active discussion, including how to build a diverse student body, a topic we do not address here.

It is important to note that the signatures that we detected are not directly biased by gender or socio-demographic status (gender as a feature was not significantly different across the signatures (Fig 2b) and race was intentionally excluded), though there is considerable work to be done to fully characterize the degree to which existing biases are persist in algorithms like the one described [32]. Interestingly, our signatures are defined primarily based upon academic metrics as opposed to non-academic metrics such as leadership or athletics experience, suggesting that these may have sufficient resolution for this purpose. However, our key substantive finding that we can successfully identify as a distinguishing signal an applicant’s capacity to overcome undergraduate academic challenges, and that that capacity is indeed predictive of future quantitative success, is a hopeful suggestion that such algorithms can be used for more informed support of individuals for future academic success. In particular, while we report different levels of performance across the signatures (Fig 3a), we also find considerable intra-signature variation (Fig 1b), suggesting that an individual student may achieve top medical school performance regardless of their signature (Fig 3c). Thus, our data-driven approach for characterizing student individuality has implications for the building of diverse communities of students and the implementation of personalized education for more successful student training. It also has the added benefit that it lends itself to automated deployment. This study, in summary, complete with its limitations and its clear potential, raises important questions for discussion in health professions education both in terms of the types of signatures identified and the algorithmic process used.

## Supporting information

S1 Table Features for the 8-year cohort. No significant difference (*P* < 0.05) was observed in the variables between the training and test groups.
(DOCX)

S2 Table Outcome data for the 8-year cohort. No significant difference (*P* < 0.05) was observed in the variables between the training and test groups.
(DOCX)

S1 Data Complete dataset. Raw data that was used for this research. A second spreadsheet contain the means and standard deviations of all features and outcome variables, across signatures.
(XLSX)

S1 Fig Squared distances from the centroids according to number of clusters. For each cluster number the sum of squared distances from the centroids is indicated by the plot. In this work, we selected four clusters and note that for this amount the second differential on this plot is greatest.
(TIF)

S1 Supplementary Note Extended methods. (DOCX)
