# Supplementary material for: Signatures of medical student applicants and academic success
Source: PLoS One. 2020 Jan 15;15(1):e0227108. doi: 10.1371/journal.pone.0227108 (PMC6961867; doi:10.1371/journal.pone.0227108)
Supplement: S1 Table — No significant difference (P < 0.05) was observed in the variables between the training and test groups. (DOCX) [file pone.0227108.s001.docx]

| **Features** | **Training group**  **(N = 851)**  **Mean (SD)** | **Test group**  **(N = 95)**  **Mean (SD)** | **Absolute**  **Cohen's d** | **Overall**  **(N = 946)**  **Mean (SD)** |
| --- | --- | --- | --- | --- |
| **Application year** | 2009.451 (2.260) | 2009.063 (2.192) | 0.172 | 2009.412 (2.255) |
| **BCPM uGPA** | 3.748 (0.201) | 3.712 (0.219) | 0.179 | 3.744 (0.203) |
| **BCPM classes hours** | 62.783 (14.398) | 61.802 (12.373) | 0.069 | 62.684 (14.205) |
| **Total uGPA** | 3.773 (0.156) | 3.755 (0.164) | 0.117 | 3.771 (0.157) |
| **Total uGPA improved –**  **Sophomore year** | 0.548 (0.498) | 0.516 (0.502) | 0.064 | 0.544 (0.498) |
| **Total uGPA improved –**  **Junior year** | 0.633 (0.482) | 0.632 (0.485) | 0.004 | 0.633 (0.482) |
| **BCPM uGPA improved –**  **Sophomore year** | 0.445 (0.497) | 0.442 (0.499) | 0.007 | 0.445 (0.497) |
| **BCPM uGPA improved –**  **Junior year** | 0.539 (0.499) | 0.463 (0.501) | 0.153 | 0.532 (0.499) |
| **Did postbac** | 0.028 (0.166) | 0.011 (0.103) | 0.110 | 0.026 (0.160) |
| **Attended grad school** | 0.026 (0.159) | 0.042 (0.202) | 0.099 | 0.027 (0.164) |
| **BCPM A's** | 15.231 (5.630) | 13.800 (4.971) | 0.257 | 15.088 (5.582) |
| **BCPM B's** | 3.700 (3.171) | 3.863 (2.959) | 0.052 | 3.717 (3.150) |
| **BCPM C's** | 0.214 (0.589) | 0.316 (0.733) | 0.168 | 0.224 (0.606) |
| **BCPM D's** | 0.002 (0.048) | 0.011 (0.103) | 0.145 | 0.003 (0.056) |
| **BCPM F's** | 0.001 (0.034) | 0.000 (0.000) | 0.036 | 0.001 (0.033) |
| **Math A's** | 1.811 (1.487) | 1.537 (1.253) | 0.187 | 1.783 (1.467) |
| **Math B's** | 0.424 (0.713) | 0.516 (0.756) | 0.128 | 0.433 (0.717) |
| **Math C's** | 0.042 (0.207) | 0.053 (0.268) | 0.048 | 0.043 (0.214) |
| **Math D's** | 0.001 (0.034) | 0.000 (0.000) | 0.036 | 0.001 (0.033) |
| **Math F's** | 0.000 (0.000) | 0.000 (0.000) | 0.000 | 0.000 (0.000) |
| **Biology A's** | 5.649 (3.670) | 5.242 (2.898) | 0.113 | 5.608 (3.601) |
| **Biology B's** | 1.298 (1.486) | 1.463 (1.457) | 0.111 | 1.315 (1.484) |
| **Biology C's** | 0.069 (0.293) | 0.095 (0.359) | 0.085 | 0.072 (0.300) |
| **Biology D's** | 0.000 (0.000) | 0.011 (0.103) | 0.324 | 0.001 (0.033) |
| **Biology F's** | 0.001 (0.034) | 0.000 (0.000) | 0.036 | 0.001 (0.033) |
| **Chemistry A's** | 5.452 (3.048) | 4.958 (2.721) | 0.164 | 5.403 (3.019) |
| **Chemistry B's** | 1.463 (1.565) | 1.400 (1.410) | 0.041 | 1.457 (1.549) |
| **Chemistry C's** | 0.082 (0.326) | 0.147 (0.412) | 0.194 | 0.089 (0.336) |
| **Chemistry D's** | 0.001 (0.034) | 0.000 (0.000) | 0.036 | 0.001 (0.033) |
| **Chemistry F's** | 0.000 (0.000) | 0.000 (0.000) | 0.000 | 0.000 (0.000) |
| **Physics A's** | 2.320 (1.570) | 2.063 (1.295) | 0.166 | 2.294 (1.546) |
| **Physics B's** | 0.515 (0.805) | 0.484 (0.797) | 0.038 | 0.512 (0.804) |
| **Physics C's** | 0.020 (0.140) | 0.021 (0.144) | 0.008 | 0.020 (0.140) |
| **Physics D's** | 0.000 (0.000) | 0.000 (0.000) | 0.000 | 0.000 (0.000) |
| **Physics F's** | 0.000 (0.000) | 0.000 (0.000) | 0.000 | 0.000 (0.000) |
| **MCAT total percentile** | 90.541 (8.190) | 88.677 (9.445) | 0.224 | 90.354 (8.338) |
| **MCAT Biology percentile** | 90.406 (9.017) | 88.228 (11.798) | 0.233 | 90.187 (9.349) |
| **MCAT Physics-Chemistry percentile** | 89.803 (10.244) | 87.466 (12.428) | 0.223 | 89.568 (10.500) |
| **MCAT count** | 1.152 (0.378) | 1.168 (0.376) | 0.045 | 1.153 (0.378) |
| **Parents education score** | 4.331 (2.251) | 4.463 (2.466) | 0.058 | 4.345 (2.273) |
| **Median income zipcode** | 88502.629 (36593.070) | 88218.611 (39919.835) | 0.008 | 88474.107 (36918.321) |
| **Gender** | 0.496 (0.500) | 0.537 (0.501) | 0.082 | 0.500 (0.500) |
| **Age at application** | 21.622 (1.498) | 21.526 (1.165) | 0.065 | 21.612 (1.468) |
| **Top 25 college or advanced degree** | 0.524 (0.500) | 0.484 (0.502) | 0.080 | 0.520 (0.500) |
| **Disadvantaged** | 0.056 (0.231) | 0.063 (0.245) | 0.029 | 0.057 (0.232) |
| **Advanced degree** | 0.038 (0.190) | 0.042 (0.202) | 0.024 | 0.038 (0.191) |
| **Number of schools accepted to** | 3.329 (1.829) | 3.358 (1.725) | 0.016 | 3.332 (1.818) |
| **Publications experience** | 0.187 (0.390) | 0.126 (0.334) | 0.157 | 0.181 (0.385) |
| **Leadership experience** | 0.461 (0.499) | 0.442 (0.499) | 0.037 | 0.459 (0.499) |
| **Athletics experience** | 0.161 (0.368) | 0.137 (0.346) | 0.066 | 0.159 (0.365) |
| **Medical experience** | 0.898 (0.303) | 0.884 (0.322) | 0.044 | 0.896 (0.305) |
| **Military experience** | 0.005 (0.068) | 0.011 (0.103) | 0.080 | 0.005 (0.073) |
| **Research experience** | 0.931 (0.254) | 0.884 (0.322) | 0.178 | 0.926 (0.262) |

**S1 Table. Features for the 8-year Cohort.** No significant difference (*P* < 0.05) was observed in the variables between the training and test groups.
