# Supplementary material for: Signatures of medical student applicants and academic success
Source: PLoS One. 2020 Jan 15;15(1):e0227108. doi: 10.1371/journal.pone.0227108 (PMC6961867; doi:10.1371/journal.pone.0227108)
Supplement: S2 Table — No significant difference (P < 0.05) was observed in the variables between the training and test groups. (DOCX) [file pone.0227108.s002.docx]

| **Outcomes** | **Training group**  **(N = 851)**  **Mean (SD)** | **Test group**  **(N = 95)**  **Mean (SD)** | **Absolute**  **Cohen's d** | **Overall**  **(N = 946)**  **Mean (SD)** |
| --- | --- | --- | --- | --- |
| **Clerkship honors** | 4.309 (2.503) | 4.200 (2.327) | 0.044 | 4.298 (2.485) |
| **AOA** | 0.176 (0.381) | 0.168 (0.376) | 0.021 | 0.175 (0.381) |
| **Clerkship pass** | 0.703 (1.064) | 0.653 (0.976) | 0.047 | 0.698 (1.055) |
| **Step 1 score** | 239.397 (16.485) | 236.463 (19.531) | 0.174 | 239.103 (16.827) |
| **Step 2 score** | 246.215 (15.892) | 244.547 (17.934) | 0.104 | 246.048 (16.106) |
| **Clerkship fails** | 0.028 (0.294) | 0.000 (0.000) | 0.101 | 0.025 (0.279) |
| **Shelf exams above 90** | 5.237 (1.183) | 5.158 (1.432) | 0.066 | 5.229 (1.210) |
| **Shelf exams below 65** | 0.165 (0.493) | 0.221 (0.549) | 0.113 | 0.170 (0.499) |

**S2 Table. Outcome data for the 8-year cohort.** No significant difference (*P* < 0.05) was observed in the variables between the training and test groups.
