# Supplementary figures and images for: Signatures of medical student applicants and academic success
Source: PLoS One. 2020 Jan 15;15(1):e0227108. doi: 10.1371/journal.pone.0227108 (PMC6961867; doi:10.1371/journal.pone.0227108)

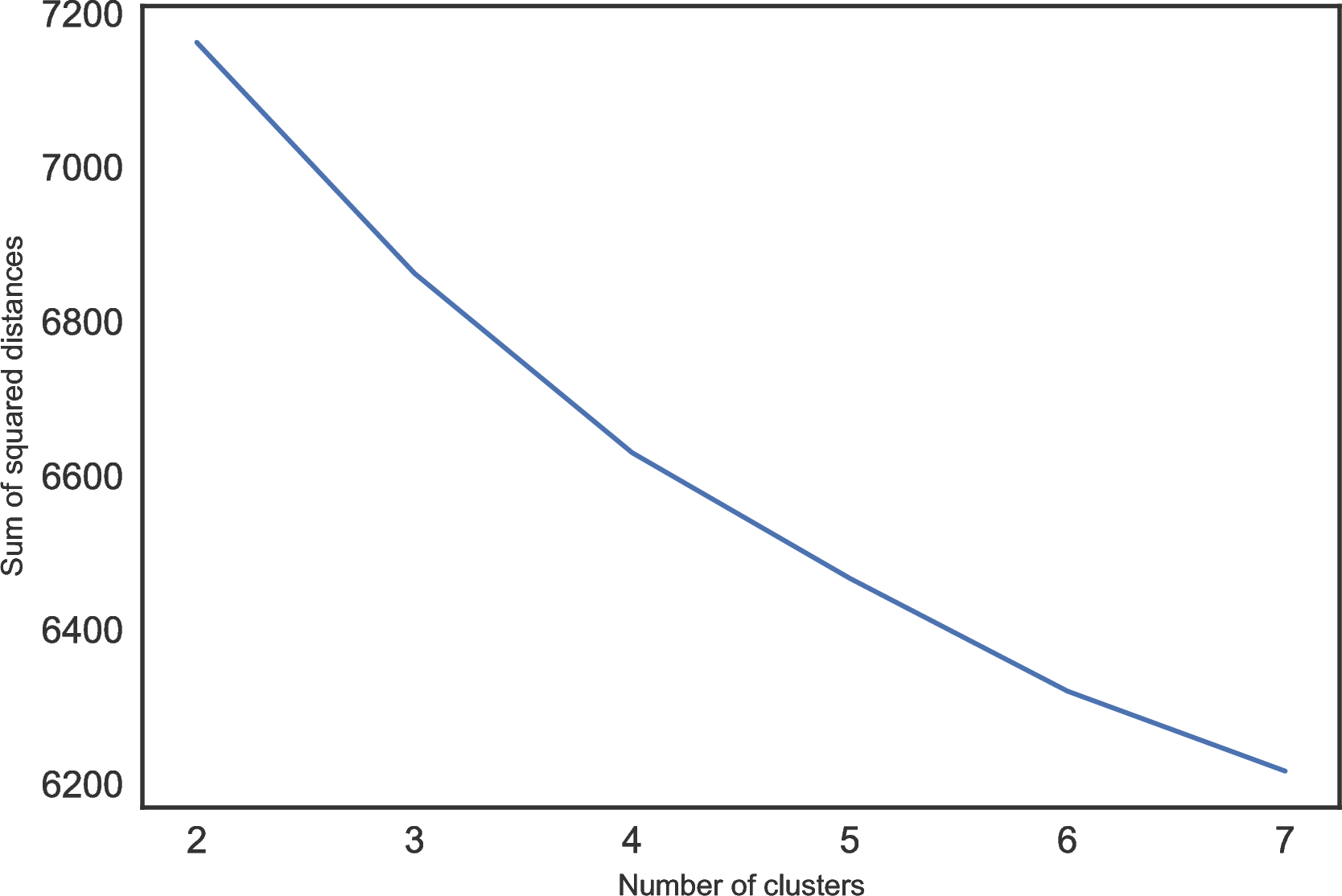

Supplement: S1 Fig — For each cluster number the sum of squared distances from the centroids is indicated by the plot. In this work, we selected four clusters and note that for this amount the second differential on this plot is greatest. (TIF) [file pone.0227108.s004.tif]
