# Supplementary material for: Signatures of medical student applicants and academic success
Source: PLoS One. 2020 Jan 15;15(1):e0227108. doi: 10.1371/journal.pone.0227108 (PMC6961867; doi:10.1371/journal.pone.0227108)
Supplement: S1 Supplementary Note — (DOCX) [file pone.0227108.s005.docx]

**Supplementary Note**

**Student application records and medical school outcomes.** We retrieved the American Medical College Application Service (AMCAS) application records, the NYU School of Medicine specific secondary application records, as well as outcomes from NYU School of Medicine for 1,088 students who applied to NYU School of Medicine between the years 2006-2014, and subsequently matriculated. Overall, 53 features and 8 informative outcomes, are available for each student (S1 Data). Out of the 1,088 students, 949 had data with no missing values, 138 had some missing outcome data and one student had missing MCAT-total-percentile data.

**Data dichotomization and computing success index.** Student data is comprised of both continuous and binary features. Using both data types together in a clustering model resulted in one of the following: 1. If the data was not scaled in any way, non-binary features, usually the high-variance features, were predominant in the model. 2. If the data was scaled, binary features were predominant in the model. To receive a model that is not biased to a specific data type, all variables (features and outcomes) were dichotomized in the following way – for every continuous variable and for every application year, the median of the variable in our dataset in a given year was calculated. If a value is lower or equal to the median it was set to zero, otherwise it was set to one. Binary variables were not altered. A variable was excluded if it had no variance in a given year, since dichotomization could not be executed. This resulted in 40 binary features and 7 binary outcomes. Student outcomes were dichotomized to allow for the composition of an aggregated success index, that is based on all available outcomes – binary and continuous. The success index was calculated as the sum of all 7 binary outcomes, resulted in a score ranging from 0 (worst) to 7 (best).

**Training-Test split.** Out of the 949 students with complete dataset, only three were from the 2014-application-year (the rest were filtered out due to missing outcome data). Since our dichotomization procedure was done based on the median of features in each year, and due to the fact that three is an unrepresentative number of students, we filtered out the three remaining 2014-application-year students. From the 946 students, 10% were randomly selected (n = 95) and set aside from the dataset to later test the validity and strength of the predictive model. This group of students was referred to as the ‘test group’, whereas the rest of the students with no missing values (n = 851) were used to train the success-prediction model, and were referred to as the ‘training group’. The selection of the 10% was stratified to maintain similar success index distributions across the training and test groups. The training group comprised from 90% of students due to the relatively small number of students with available data and the high dimensionality of the dataset, and with intentions to produce a robust model. The clustering model relies solely on admissions data and not outcome data, and our feature selection algorithm (described below) did not choose the feature MCAT-total-percentile as a predictor for the clustering model. This allowed us to add the 142 students that were filtered out to the training group, and create the ‘clustering training group’ (n = 993), that will be used to build the clustering model.

**Feature selection.** In order to improve the distinction among clusters, a feature selection approach was used to reduce the dimensionality of the clustered data, and to increase the coherence of the clusters. To determine which features of the 40 binary features to include in the clustering model, we defined an index score to indicate the combination of features that reveal the most resolved clusters. The index score comprised of the following elements: Silhouette score (*sil*) – to score the tightness of the clusters; entropy (*ent*) – to score the balance of clusters sizes; mean variance of the selected features (*var*) – to score the amount of variation accounted for by the set of features, and redundancy (*cor*) – to score the highest absolute correlation among two features in the selected features.

The feature selection algorithm receives as input: *D*, a matrix of the clustering training group, with all 40 binary features; *C*, absolute-value correlation coefficient matrix among all features; *V*, variances vector, where each feature variance is scaled to a range of [-1,1]; *P*, a vector with 40 entries, initialized to 0; *N*, a vector with 40 entries, initialized to 0; *n*, the number of tested subset of features, where *n* is an integer ranges from 1 to 40. The algorithm first creates a list of all possible subsets of features the size of *n*, shuffles the list to avoid order effects, and iterates over the subsets of features. For each subset, a K-means clustering is performed on the clustering training data containing only the features in the current tested subset of features. The silhouette score (*sil*) and entropy (*ent*) for the specific clustering are computed along with the mean variance of the subset of features (*var*), taken from *V*, and the maximum absolute correlation among two features in the subset of features (*cor*), taken from *C*. An index score is computed according to the formula: (0.5**sil*) + (0.4**ent*) + (0.3**var*) - (0.2**cor*). If this score is higher than the current maximum index score, a 1 is added to all of the entries in *P*, corresponding to the current subset of features, and the maximum index score is set to the current index score. Alternatively, if the computed index score is lower than the current minimum index score, a 1 is added to all of the entries in *N*, corresponding to the current subset of features, and the minimum index score is set to the current index score. The algorithm runs for *n* = [1, 6]. At the end of all 6 runs, we subtracted *N* from *P*, to arrive at a ranking vector, indicating for every feature if it is generally improving the clustering (high rank) or disrupting (low rank). By choosing all positive ranked features, this approach resolved in a reduced dimensionality of the feature space to 23 informational features for cluster analysis.

**K-means clustering and t-SNE visualization.** We implemented K-means clustering using the scikit-learn Python package (using parameters max_iter = 10000, n_init = 1000, tol = 0.0000001, random_state = 1) and clustered the 993 students with 23 binary features into four clusters. As a visualization technique to the 23-dimensional clustered data, we used Python’s scikit-learn implementation of t-distributed Stochastic Neighbor Embedding. The t-SNE parameters were: method = 'exact', n_iter = 5000, n_iter_without_progress = 500, learning_rate = 75, random_state = 1.

**Statistical considerations.** To determine the distinction of the clusters, we developed a bootstrapping statistical test. The bootstrap test was performed only on the training group subset of students with all features and outcomes available (n = 851). For each cluster, we computed the mean of each feature and outcome for the respective students. We then selected with replacement a random group of students (the same size as the cluster) from our total sample of 851 students. We computed the mean of each feature and outcome for the respective students in the randomly selected cluster. We repeated this procedure 100,000 times, and receive a distribution of means for each feature and outcome. The confidence interval (CI) for the difference between a cluster mean and the bootstrap distribution is calculated for each randomly selected cluster and for each variable (feature or outcome). The 95% CI is then obtained by taking the range of differences between the 2.5 and 97.5 percentiles. The *P*-value for each variable of a cluster is computed compare to the respective distribution of means for that variable. The *P*-values received in this test were transformed to –log_10_(*P*-value), indicating whether the mean value of a variable in the cluster is higher than the bootstrap distribution mean (positive values, higher value indicates lower *P*-value), or lower than the bootstrap distribution mean (negative values, higher absolute value indicates lower *P*-value).

**Inferring signatures for the test group and predicting success**. Using the fitted K-means model, we used the *predict* method to infer the appropriate signature of each student in the test group. The *predict* method assigned each student in the test group to the cluster with the closest center. In order to produce a valid and robust predictive model, and due to the relatively small sample size (n = 851) compared to the relatively wide-range of outcome (eight-level success index), we further compressed the success index into a three-level score. This was done by scoring low performing students (original success index scores of 0, 1 and 2) with 0, medium performing students (original success index scores of 3 and 4) with 1, and high performing students (original success index scores of 5, 6 and 7) with 2. We then fitted a logistic regression model, implemented by scikit-learn and optimized by a grid search, also implemented by scikit-learn, using a 3-fold cross validation procedure on the training group. The optimized predictive model was trained on the training group with 851 students and 40 dichotomized features. The target variable for the model was the three-level success index. The parameters of the chosen logistic regression model, that were altered compare to scikit-learn’s naive implementation of logistic regression were: C = 0.1, max_iter = 100000, multi_class = 'multinomial', solver = 'newton-cg'. We then tested the predictive model on the test group. To examine whether the accuracy improves upon inclusion of the signature information, we fitted a second logistic regression model, that was trained on the same cohort of 851 students, with the sole change that the signature of the student is included as an additional feature. The target variable for this second model was also the three-level success index. The parameters of the chosen logistic regression model, that were altered compare to scikit-learn’s naive implementation of logistic regression were: C = 0.1, max_iter = 100000, multi_class = 'multinomial', solver = 'newton-cg'. We then tested the predictive model on the test group. The Likelihood Ratio (LR) test was performed on the two fitted models using the training data.
